# Repeatability and Reproducibility of Intraocular Pressure and Dynamic Corneal Response Parameters Assessed by the Corvis ST

**DOI:** 10.1155/2017/8515742

**Published:** 2017-06-06

**Authors:** Bernardo T. Lopes, Cynthia J. Roberts, Ahmed Elsheikh, Riccardo Vinciguerra, Paolo Vinciguerra, Sven Reisdorf, Stefanie Berger, Robert Koprowski, Renato Ambrósio

**Affiliations:** ^1^Rio de Janeiro Corneal Tomography and Biomechanics Study Group, Rio de Janeiro, RJ, Brazil; ^2^Department of Ophthalmology, Federal University of São Paulo, São Paulo, SP, Brazil; ^3^Department of Ophthalmology & Visual Science and Department of Biomedical Engineering, The Ohio State University, Columbus, OH, USA; ^4^School of Engineering, University of Liverpool, Liverpool, UK; ^5^Department of Surgical Sciences, Division of Ophthalmology, University of Insubria, Varese, Italy; ^6^Eye Center, Humanitas Clinical and Research Center, Via Manzoni 56, Rozzano, Italy; ^7^Humanitas University, Via Manzoni 56, Rozzano, Italy; ^8^Vincieye Clinic, Milan, Italy; ^9^OCULUS Optikgeräte GmbH, Wetzlar, Germany; ^10^Department of Biomedical Computer Systems, Faculty of Computer Science and Materials Science, Institute of Computer Science, University of Silesia, Bedzinska 39, 41-200 Sosnowiec, Poland

## Abstract

**Purpose:**

To assess the repeatability and reproducibility of dynamic corneal response parameters measured by the Corvis ST (Oculus, Wetzlar, Germany).

**Methods:**

One eye randomly selected from 32 healthy volunteers was examined by the Corvis ST. Three different devices were used in an alternated random order for taking three measurements at each device in each subject. Standard intraocular pressure (IOP), the biomechanical-compensated IOP (bIOP), and DCR parameters were evaluated. The within-subject standard deviation (*ζ*w) and coefficient of variation (CV) were assessed.

**Results:**

Regarding pressure indices, the *ζ*w was below 1 mmHg for repeatability (0.98 for IOP and 0.89 for bIOP) and the CV was 6.6% for IOP and 6.1% for bIOP. For reproducibility, the *ζ*w was around 1 mmHg (1.12 for IOP and 1.05 for bIOP) and the CV was 7.6% for IOP and 7.1% for bIOP. Most of DCR indices presented CV for repeatability below 4%. For reproducibility, the CV of most of the indices were below 6%. The deformation amplitude (DA) ratio in 1 mm and integrated radius were below 4% (1.2% and 3.8%, resp.).

**Conclusions:**

The Corvis ST showed good precision (repeatability and reproducibility) for IOP measurements and for DCR in healthy eyes.

## 1. Introduction

Corneal biomechanical assessment has an important role for the diagnosis and characterization of ocular diseases such as keratoconus, Fuch's dystrophy, and glaucoma [1–3]. Biomechanical fragility is also related to the susceptibility of the cornea to ectasia progression, which is an ultimate factor for assessing the risk for iatrogenic kerectasia after laser vision correction [4–6]. In addition, therapeutic manipulation of corneal biomechanics has been introduced as a treatment for ectatic corneal diseases [7] and other ocular conditions such as presbyopia [8].

In vivo corneal biomechanics assessment started in 2005 with the introduction of the Ocular Response Analyzer (ORA; Reichert Ocular Instruments, Depew, NY) [9]. The ORA combines an air puff with an infrared light emitter and receiver. This device only allows an indirect assessment of the corneal deformation based on the signal of the infrared light. The Corvis ST (OCULUS Optikgeräte Inc., Wetzlar, Germany) is a relatively new corneal biomechanics device, composed of an air puff indentation system and ultra-high-speed Scheimpflug technology. The camera has a blue-light LED and acquires a sequence of 140 images of the deformation process at over 4330 frames/s with 8 mm horizontal coverage. With this technology, it is possible to actually see how the cornea deforms in response to the air puff pressure [10].

The new software of the Corvis ST provides new parameters based on corneal deformation [11, 12]. The present study examines the repeatability and reproducibility of these new parameters in normal corneas.

## 2. Methods

The study was conducted in healthy volunteers, conforming to the tenets of the Declaration of Helsinki, and was approved by the ethical committee. The study included thirty two volunteers with normal ophthalmic examinations. Exclusion criteria were the presence of any corneal disease, history of ocular surgery or trauma, contact lens wear, pregnancy, or other ocular conditions different than refractive error. One eye randomly selected from each participant was chosen. Each eye was examined by an experienced technician using three different Corvis ST devices, three times in each device. The measurements were taken alternately in each device in a random order in order to estimate between instrument variability and total reproducibility.

We analyzed the intraocular pressure (IOP) provided by the Corvis ST, the biomechanical-compensated IOP (bIOP) [11, 13], and the dynamic corneal response (DCR) parameters: maximum deformation amplitude (DA Max), maximum deflection amplitude (DefA Max), DA ratio in 2 mm [12] and DA ratio in 1 mm, integrated radius, maximum inverse radius, the first applanation (A1) velocity, and stiffness parameter at the first applanation (SP A1).

An ANOVA model was used to assess the repeatability and reproducibility. It was built with a random subject, a random device, and random interactions between subjects and devices as factors.

Y*ijk* = *μ* + S*i* + M*j* + SM*ij* + E*ijk* with subject *i* = 1.32; device *j*=1, 2, and 3; repeat *k* = 1, 2, and 3.

Repeatability of measurements refers to the variation in repeat measurements made on the same subject under identical conditions. Reproducibility refers to the variation in measurements made on a subject under changing conditions, in this case the different devices [14]. Within-subject standard deviation (*ζ*w), coefficient of variation (CV), and coefficient of repeatability (CR) were calculated from the random-effects model. The CV is defined as the ratio of *ζ*w to the overall mean. A lower CV is closely related to higher repeatability or reproducibility. The CR is the √2 × 1.96 *ζ*w or 2.77 × *ζ*w. The difference between two measurements for the same subject is expected to be less than 2.77 *ζ*w for 95% of pairs of observations.

Statistical analysis was accomplished with R Core Team (2016), a language and environment for statistical computing (R Foundation for Statistical Computing, Vienna, Austria, URL: https://www.R-project.org/).

## 3. Results

The male : female rate was 1 : 1. The mean age was 37.3 ± 11.7, ranging from 18.6 to 64.2 years.

Table 1 shows the values of *ζ*w, CV, and CR for repeatability and reproducibility derived from the random-effects model for IOP, bIOP, and DCRs.

Considering the pressure indices, the *ζ*w was below 1 mmHg for repeatability (0.98 for IOP and 0.89 for bIOP) and the CV and CR were 6.6% and 2.7 for IOP and 6.1% and 2.4 for bIOP, respectively. For reproducibility, the *ζ*w was around 1 mmHg (1.12 for IOP and 1.05 for bIOP) and the CV and CR were 7.6% and 3.1 for IOP and 7.1% and 2.9 for bIOP, respectively.

Most of DCR indices presented CV for repeatability below 4%. A1 velocity and SP A1 had slightly higher CV, 5.4% and 5%, respectively. For reproducibility, the CV of most of the indices was below 6%. DAratio in 1 mm and integrated radius were below 4% (1.2% and 3.8%, resp.). A1 velocity and SP A1 were slightly higher (7.9% and 6.5%, resp.).

## 4. Discussion

The Corvis ST allowed a new perspective for the measurement of corneal biomechanics. The parameters obtained with the device have presented good reliability in virgin and post-PRK eyes [15]. Repeatability was also good in normal and in keratoconic eyes [16]. New indices of DCRs have been developed and are showing good results in demonstrating biomechanical fragility of the keratoconic cornea [17]. They are part of a new display in the device, developed with a software upgrade in processing the signals. Since this is relatively new equipment, there are few studies assessing repeatability and reproducibility of its measures. To the best of our knowledge, this is the first study to investigate the precision of these new variables. In this study, we aimed to assess the repeatability and reproducibility of these new indices, along with IOP and bIOP.

In our study, the repeatability and reproducibility (*ζ*w) of IOP were very good, approximately 1 mmHg (0.98 and 1.12, resp.). The CV was 6.6% for repeatedly and 7.6% for reproducibility, and the CR were also low, below 3 mmHg for repeatability and around 3 mmHg for reproducibility. This is consistent with previous studies. Nemeth et al. found CV of 6.9% for the IOP repeatability [18]. Ali et al. found similar results to IOP repeatability with CV of 6.1% [19]. Bak-Nielsen et al. assessed not just repeatability but also reproducibility with measurements in different days [20]. In their study, they found slightly lower values of CV, 4.2% for repeatability and 6.5% for reproducibility.

The bIOP is obtained with a method to measure the IOP in a way that it is less influenced by the stiffness of the cornea [13]. In ex vivo human eye globes, the bIOP was the closest measure to the true IOP. In in vivo studies, it was less associated with corneal thickness and age [11]. The repeatability and reproducibility of this pressure in our study were similar to the IOP around 1 mmHg (0.89 and 1.05, resp.). The CV was 6.1% and 7.2% and the CR was 2.4 and 2.9 for repeatability and reproducibility, respectively.

The DCRs presented good precision. The CV of repeatability and reproducibility for most of the indices were below 4% and 6%, respectively.

One of the first aspects that is noticed in the exam is the maximum amplitude of corneal deformation. It presented good repeatability, CV of 3.8%, and reproducibility, CV of 5.7%. It is consistent with other studies where the CV for repeatability ranged from 3.64% to 4.3% [18–20].

When we correct the maximum deformation amplitude for the whole eye movement, we obtain the maximum deflection amplitude, which presented also good repeatability, CV of 3.7%, and reproducibility, CV of 5.3%. Bak-Nielsen et al. had also investigated the precision of this variable and found similar results for repeatability, CV of 4.4%, and reproducibility, CV of 4.2%.

Five other new variables analyzed in this study (DAratio in 2 mm, DAratio in 1 mm, integrated radius, maximum inverse radius, and SP A1) presented good precision [20]. The first four presented repeatability CV less than 4% and the reproducibility CV less than 5%. The SP A1 presented slightly higher repeatability and reproducibility CV (5% and 6.5%, resp.); this can be explained by the fact that it is a complex parameter that combines several information provided by the device.

The A1 velocity was the DCR variable with higher repeatability and reproducibility CV (5.4% and 7.9%, resp.). In previous studies, the repeatability CV were much higher, ranging from 14.8% to 17.1% [18–20]. One study assessed the reproducibility CV and found also a higher value (13.5%) [20]. The difference in the precision of this variable in our study was due to the new software that uses a Gaussian smoothing algorithm and allows more reliable measures of applanation velocity.

## 5. Conclusion

The Corvis ST showed good precision (repeatability and reproducibility) for IOP measurements and for DCR parameters in healthy eyes.

## Figures and Tables

**Table 1:** Corvis ST repeatability and reproducibility IOP and DCR indices.

	Mean	Repeatability			Reproducibility		
		*ζ*w	CV	CR	*ζ*w	CV	CR
IOP (mmHg)	14.73	0.98	0.066	2.7146	1.12	0.076	3.1024
bIOP(mmHg)	14.57	0.89	0.061	2.4653	1.05	0.072	2.9085
DA Max (mm)	1.09	0.04	0.038	0.1108	0.06	0.057	0.1662
DefA Max (mm)	0.91	0.03	0.037	0.0831	0.04	0.053	0.1108
DAratio in 2 mm	4.29	0.13	0.032	0.3601	0.23	0.054	0.6371
DAratio in 1 mm	1.57	0.019	0.012	0.05263	0.025	0.012	0.06925
Integrated radius (mm^−1^)	8.17	0.26	0.031	0.7202	0.31	0.038	0.8587
Maximum inverse radius (mm^−1^)	0.139	0.003	0.024	0.00831	0.006	0.046	0.01662
A1 velocity (m/s)	0.15	0.008	0.054	0.02216	0.012	0.079	0.03324

IOP: intraocular pressure; bIOP: biomechanical-corrected IOP; DA Max: maximum deformation amplitude; DefA Max: maximum deflection amplitude; DAratio: deformation amplitude ratio; integrated radius: integrated sum of inverse radius between the first and second applanation events; maximum inverse radius: inverse concave radius at the highest concavity moment; A1 velocity: speed of the corneal apex at applanation.
